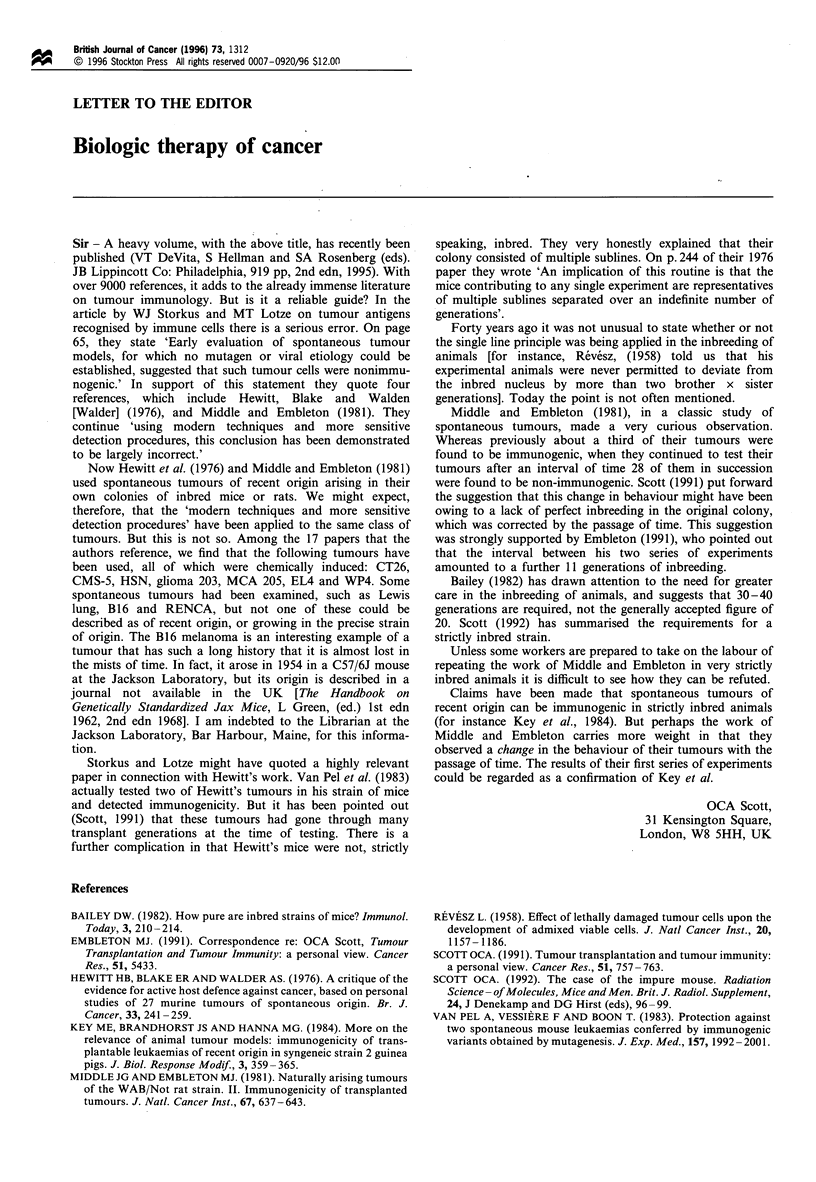# Biologic therapy of cancer.

**DOI:** 10.1038/bjc.1996.250

**Published:** 1996-05

**Authors:** O. C. Scott


British Journal of Cancer (1996) 73, 1312
© 1996 Stockton Press All rights reserved 0007-0920/96 $12.00

LETTER TO THE EDITOR

### Biologic therapy of cancer

**Sir** – A heavy volume, with the above title, has recently been published (VT DeVita, S Hellman and SA Rosenberg (eds). JB Lippincott Co: Philadelphia, 919 pp, 2nd edn, 1995). With over 9000 references, it adds to the already immense literature on tumour immunology. But is it a reliable guide? In the article by WJ Storkus and MT Lotze on tumour antigens recognised by immune cells there is a serious error. On page 65, they state 'Early evaluation of spontaneous tumour models, for which no mutagen or viral etiology could be established, suggested that such tumour cells were nonimmunogenic.' In support of this statement they quote four references, which include Hewitt, Blake and Walden [Walder] (1976), and Middle and Embleton (1981). They continue 'using modern techniques and more sensitive detection procedures, this conclusion has been demonstrated to be largely incorrect.'

Now Hewitt *et al.* (1976) and Middle and Embleton (1981) used spontaneous tumours of recent origin arising in their own colonies of inbred mice or rats. We might expect, therefore, that the 'modern techniques and more sensitive detection procedures' have been applied to the same class of tumours. But this is not so. Among the 17 papers that the authors reference, we find that the following tumours have been used, all of which were chemically induced: CT26, CMS-5, HSN, glioma 203, MCA 205, EL4 and WP4. Some spontaneous tumours had been examined, such as Lewis lung, B16 and RENCA, but not one of these could be described as of recent origin, or growing in the precise strain of origin. The B16 melanoma is an interesting example of a tumour that has such a long history that it is almost lost in the mists of time. In fact, it arose in 1954 in a C57/6J mouse at the Jackson Laboratory, but its origin is described in a journal not available in the UK [*The Handbook on Genetically Standardized Jax Mice*, L Green, (ed.) 1st edn 1962, 2nd edn 1968]. I am indebted to the Librarian at the Jackson Laboratory, Bar Harbour, Maine, for this information.

Storkus and Lotze might have quoted a highly relevant paper in connection with Hewitt's work. Van Pel *et al.* (1983) actually tested two of Hewitt's tumours in his strain of mice and detected immunogenicity. But it has been pointed out (Scott, 1991) that these tumours had gone through many transplant generations at the time of testing. There is a further complication in that Hewitt's mice were not, strictly speaking, inbred. They very honestly explained that their colony consisted of multiple sublines. On p. 244 of their 1976 paper they wrote 'An implication of this routine is that the mice contributing to any single experiment are representatives of multiple sublines separated over an indefinite number of generations'.

Forty years ago it was not unusual to state whether or not the single line principle was being applied in the inbreeding of animals [for instance, Révész, (1958) told us that his experimental animals were never permitted to deviate from the inbred nucleus by more than two brother × sister generations]. Today the point is not often mentioned.

Middle and Embleton (1981), in a classic study of spontaneous tumours, made a very curious observation. Whereas previously about a third of their tumours were found to be immunogenic, when they continued to test their tumours after an interval of time 28 of them in succession were found to be non-immunogenic. Scott (1991) put forward the suggestion that this change in behaviour might have been owing to a lack of perfect inbreeding in the original colony, which was corrected by the passage of time. This suggestion was strongly supported by Embleton (1991), who pointed out that the interval between his two series of experiments amounted to a further 11 generations of inbreeding.

Bailey (1982) has drawn attention to the need for greater care in the inbreeding of animals, and suggests that 30–40 generations are required, not the generally accepted figure of 20. Scott (1992) has summarised the requirements for a strictly inbred strain.

Unless some workers are prepared to take on the labour of repeating the work of Middle and Embleton in very strictly inbred animals it is difficult to see how they can be refuted.

Claims have been made that spontaneous tumours of recent origin can be immunogenic in strictly inbred animals (for instance Key *et al.*, 1984). But perhaps the work of Middle and Embleton carries more weight in that they observed a *change* in the behaviour of their tumours with the passage of time. The results of their first series of experiments could be regarded as a confirmation of Key *et al.*

OCA Scott,
31 Kensington Square,
London, W8 5HH, UK

### References

BAILEY DW. (1982). How pure are inbred strains of mice? *Immunol. Today*, **3,** 210–214.

EMBLETON MJ. (1991). Correspondence re: OCA Scott, *Tumour Transplantation and Tumour Immunity*: a personal view. *Cancer Res.*, **51,** 5433.

HEWITT HB, BLAKE ER AND WALDER AS. (1976). A critique of the evidence for active host defence against cancer, based on personal studies of 27 murine tumours of spontaneous origin. *Br. J. Cancer*, **33,** 241–259.

KEY ME, BRANDHORST JS AND HANNA MG. (1984). More on the relevance of animal tumour models: immunogenicity of transplantable leukaemias of recent origin in syngeneic strain 2 guinea pigs. *J. Biol. Response Modif.*, **3,** 359–365.

MIDDLE JG AND EMBLETON MJ. (1981). Naturally arising tumours of the WAB/Not rat strain. II. Immunogenicity of transplanted tumours. *J. Natl. Cancer Inst.*, **67,** 637–643.

RÉVÉSZ L. (1958). Effect of lethally damaged tumour cells upon the development of admixed viable cells. *J. Natl Cancer Inst.*, **20,** 1157–1186.

SCOTT OCA. (1991). Tumour transplantation and tumour immunity: a personal view. *Cancer Res.*, **51,** 757–763.

SCOTT OCA. (1992). The case of the impure mouse. *Radiation Science – of Molecules, Mice and Men. Brit. J. Radiol. Supplement*, **24,** J Denekamp and DG Hirst (eds), 96–99.

VAN PEL A, VESSIÈRE F AND BOON T. (1983). Protection against two spontaneous mouse leukaemias conferred by immunogenic variants obtained by mutagenesis. *J. Exp. Med.*, **157,** 1992–2001.